# Genome Sequence of *Streptomyces* sp. Strain GQFP Isolated from Soil Near the Roots of Pharmaceutical Plant Elaeagnus pungens

**DOI:** 10.1128/mra.00002-22

**Published:** 2022-04-04

**Authors:** Jie Zhu

**Affiliations:** a State Key Laboratory of Microbial Metabolism, Joint International Research Laboratory of Metabolic & Developmental Sciences, School of Life Sciences & Biotechnology, Shanghai Jiao Tong University, Shanghai, China; University of Arizona

## Abstract

Here, we report the complete and linear genome sequence of *Streptomyces* sp. strain GQFP, isolated from the soil microbiome of the root of pharmaceutical plant Elaeagnus pungens. The strain contained one 8,306,813-bp chromosome, with a GC content of 69.8%.

## ANNOUNCEMENT

Microorganisms often play diverse and vital roles in the growth of pharmaceutical plant hosts, such as stress tolerance, resistance to pathogens, promotion of plant growth, and uptake of a diversity nutrient substances (1–4). The genomic information of individual members of microorganisms facilitated our understanding toward coassociations between individual symbiotic microorganisms and pharmaceutical plant hosts.

Here, we have isolated the microbes from the soil sticking to the root of Elaeagnus pungens, whose leaf is one of the antiasthmatic traditional Chinese medicines (5). A strain of the genus *Streptomyces* was isolated from the soil sample. The strain was cultured in ISP2 agar plates at 30°C for 7 days. The ISP2 agar plates contained 4 g/L yeast extract, 10 g/L malt extract, 4 g/L glucose (pH 7.5), and 20 g/L agar. The strain was then transferred into trypticase soy broth with yeast (TSBY) liquid medium in 250-mL flasks at 30°C and 220 rpm for 5 days. The TSBY liquid medium contained 30 g/L tryptone soya broth, 5 g/L yeast extract, and 103 g/L sucrose (pH 7.5). The mycelia were harvested through centrifugation and washed twice in distilled H_2_O. After centrifugation, the mycelia were stored in −80°C for genome sequencing.

Genomic DNA was extracted from the mycelia using the cetyltrimethylammonium bromide method (6). Sequencing of genomic DNA was conducted at Personalbio (Shanghai Personal Biotechnology Co., Ltd., Shanghai, China). To construct libraries with DNA fragments of different lengths, a whole-genome shotgun strategy was performed first. Genomic sequencing was conducted using both the Pacific Biosciences (PacBio) and Illumina HiSeq platforms. The PacBio sequencing platform produced 104,162 sequences and 905,073,920 bp of high-quality bases. PacBio sequences were assembled with HGAP4 (7) and CANU v. 1.6 (8). For Illumina sequencing, the genomic DNA was randomly fragmented to 400 bp. Through end polishing, A-tailing, and ligation with adapters, the library was sequenced on an Illumina HiSeq platform, generating 2 × 150-bp paired-end reads. Raw data were further trimmed using AdapterRemoval v. 2.1.7 (9) to remove adapters and polished using SOAPec v. 2.0 (10). The Illumina HiSeq platform generated 24,032,166 high-quality reads. Subsequently, the high-quality reads from the Illumina HiSeq platform were used to polish the assembly generated by the PacBio platform with Pilon v. 1.22 (11). The total genome was determined to comprise 8,306,813 bp with a GC content of 69.8%.

To obtain more information of the genome, further analysis was performed. A total of 62 tRNAs were predicted by tRNAscan-SE v. 1.3.1 (12). The protein-coding gene prediction was conducted using GeneMarkS v. 4.32 (13), thereby resulting in a total of 7,479 protein-coding genes with the average length of 969.24 bp. Further functional analyses of these genes were performed based on different databases, including the NCBI nonredundant (NR) database (14), Nonsupervised Orthologous Groups (NOG) (15), Kyoto Encyclopedia of Genes and Genomes (KEGG) (16), Swiss-Prot, and Gene Ontology (GO) (17). To gain insight into the secondary metabolism of the strain, antiSMASH v. 6.0 (18) was used to analyze the secondary metabolite biosynthetic gene clusters with the “bacterial taxon” option. The analyses revealed a total of 24 putative secondary metabolite biosynthetic gene clusters, which could generate siderophores (19–21), lanthipeptides (22, 23), and terpenes (24–26). Default parameters were used for all software.

The complete genome sequence revealed biological activities and potential secondary metabolites of the strain, which could provide valuable understanding toward the coassociation between the strain with the plant host.

### Data availability.

The genome sequence has been deposited in GenBank under the accession number CP089312, BioSample accession number SAMN24594572, and BioProject accession number PRJNA794000. The raw sequencing data were deposited in the SRA database under the accession number SRR18249970 and SRR18249971. The features of the genome are summarized in Table 1.

**TABLE 1:** Genome features of *Streptomyces* sp. GQFP isolated from soil near the roots of pharmaceutical plant Elaeagnus pungens

Parameter	Feature
GenBank accession no. for genome assembly	CP089312
No. of high-quality paired-end Illumina reads	24,032,166
Total sequence no. for the PacBio sequencing	104,162
Maximum sequence length of the PacBio sequencing (bp)	43,568
Minimum sequence length of the PacBio sequencing (bp)	200
*N*_50_ length of the PacBio sequencing (bp)	11,173
*N*_90_ length of the PacBio sequencing (bp)	5,303
Assembly size (bp)	8,306,813
G+C content (%)	69.8
Total gene length (bp)	7,151,034
Total no. of genes	7,479
Avg gene length (bp)	969.24
Gene percentage of genome (%)	86.09
No. of tRNA genes	62
Secondary metabolites gene clusters	24
